# Early treatment and toxicity outcomes for small posterior uveal melanomas treated using custom loaded eye plaques

**DOI:** 10.3389/fmed.2023.1055141

**Published:** 2023-05-05

**Authors:** Sara Medek, Zelia M. Correa, Brad Huth, Vinita Takiar

**Affiliations:** ^1^Department of Radiation Oncology, University of Cincinnati Medical Center, Cincinnati, OH, United States; ^2^Ocular Oncology, Bascom Palmer Eye Institute, University of Miami Health System, Miami, FL, United States; ^3^Department of Radiation Oncology, St. Elizabeth’s Hospital, Edgewood, KY, United States

**Keywords:** uveal melanoma, plaque, custom loading configuration, outcomes, toxicities

## Abstract

**Introduction:**

Iodine-125 loaded Collaborative Ocular Melanoma Study plaques can achieve excellent tumor control for patients diagnosed with uveal melanomas. Our ocular cancer team hypothesized that use of novel, partially loaded COMS plaques could ease and improve accurate plaque placement during treatment of small, posterior tumors while providing equivalent tumor control.

**Materials/methods:**

Records of 25 patients treated with custom plaques were compared to 20 patients treated with fully loaded plaques, who had received treatment prior to our institution’s adopting the use of these partial plaques. Tumors were matched with regards to location and dimensions as measured by the ophthalmologist. Retrospective analysis of dosing parameters, tumor control and toxicity outcomes were performed.

**Results:**

There were no cancer related deaths, local recurrences or metastases in either cohort at an average follow up of 24 months for patients treated with custom plaques and 60.7 months for patients treated with fully loaded plaques. No statistically significant difference was found in regards to post-operative development of cataracts (*χ*^2^ = 0.76) or radiation retinopathy (*χ*^2^ = 0.22). Patients treated with custom loaded plaques noted significantly less clinical visual loss (*χ*^2^ = 0.006) and were more likely to have vision preserved at ≥20/200 (*χ*^2^ = 0.006).

**Conclusion:**

Treatment of small, posterior uveal melanomas with partially loaded COMS plaques results in equivalent survival and recurrence outcomes as treatment with fully loaded plaques, while exposing the patient to less radiation. Additionally, treatment with partially loaded plaques reduces the incidence of clinically significant visual loss. These promising early results support the use of partially loaded plaques in well-selected patients.

## 1. Introduction

Uveal melanomas account for approximately 82.5% of all ocular melanomas and are the most common primary intraocular malignancy in adults (1). In the United States the incidence has been noted to be 5.1 per million people with a mean age at diagnosis of 58 years (2, 3). While disease can affect any part of the uveal tract, the majority of cases occur in the choroid (1). Until the 1980s the standard of care for treating uveal melanomas was enucleation. However after a series of case studies were published demonstrating the effectiveness of radiation, a multicenter prospective clinical trial was initiated to directly compare radiotherapy to enucleation (4–6). In 2001, the Collaboration Ocular Melanoma Study (COMS) demonstrated no difference in melanoma related mortality between patients undergoing enucleation or iodine 125 brachytherapy for medium and large sized tumors (7).

Despite favorable outcomes, brachytherapy is not without risk as subsequent COMS papers have documented an increase in posterior pole abnormalities, substantial decreased visual acuity and dose-related increased risk of cataract formation (8–10). There are multiple challenges associated with treating far posterior uveal melanomas with brachytherapy, particularly when tumors are small in size. One issue is the technical challenge of accurately placing a small plaque posterior to the equator of the eye. Additionally, small plaques located in this area are more likely to tilt with eye movements during the treatment interval. While larger sized plaques are easier to surgically insert and less prone to tilting, a fully loaded plaque using COMS standard design would also deliver radiation to a larger portion of the eye. Moreover, as previous studies have demonstrated, dose of radiation to the foveola and optic nerve head are important predictors for posterior pole abnormalities and therefore the use of larger fully loaded plaques is not ideal (8). Thus, alternatives to a standard COMS plaque merit investigation.

Our ocular cancer team proposed customized partial loading of the silastic COMS plaque carrier in order to deliver an appropriate radiation dose to the target lesion while facilitating placement with the use of a larger sized plaque. The creation of custom plaques has been done previously (11–13). However, our use of a silastic carrier as compared to hand placed sources is advantageous for quality assurance (QA) and confirmation of source placement with the additional dosimetric benefits of the carrier. Here we review the treatment and toxicity outcomes.

## 2. Materials and methods

### 2.1. Patients

We conducted an Institutional Review Board (IRB) approved, retrospective review of patients treated with custom loaded eye plaques as compared to similar fully loaded plaques. Patients selected for treatment with custom loaded plaques had small, sub-centimeter tumors located such that a fully loaded plaque plus 2 mm radial margin would require suture placement posterior to the equator. Additionally, patient selection was limited to cases in which the total activity of the plaque necessary to treat the maximal depth of the tumor with an additional 1 mm thickness of the sclera could be accommodated with a limited number of sources, with individual source activity limited to 9 mCi due to manufacturing restrictions. At our institution custom loaded plaques were first used in June 2013. Therefore we identified all cases of custom eye plaque placement from 6/2013–3/2016 for a total of 29 cases. Of those cases, four were excluded for the following reasons: one was consistent with metastasis from esophageal cancer, one was located in the iris, one was a relapse of a previously treated uveal melanoma and one lacked any follow-up information post-operatively. This resulted in a total of 25 patients for evaluation in the custom eye plaque cohort.

Our comparison group was selected from the time period leading up to the initiation of custom plaques dating from 3/2009–6/2013. Selection criteria were chosen to find a similar cohort to the custom plaque patients in terms of location and tumor size. Therefore, patients were selected who had tumors completely located posterior to the equator with maximum tumor volume of ≤5 cc and a largest basal diameter of less than 9 mm. This resulted in a cohort of 20 patients in the fully loaded eye plaque group. Due to referral practices, our comparison group was selected from over a longer timeframe and remains smaller than our custom plaque cohort as these tumors were less frequently seen in our clinic.

### 2.2. Data analysis

Patients were collectively analyzed for tumor control parameters including overall survival, progression free survival, and enucleation free survival. Overall survival was defined as the number of patients alive at time of most recent follow-up. Progression free survival was noted to be the duration of time in which the patient had no evidence of intraocular recurrence, defined as an increase in size on ophthalmic evaluation, and no development of distant disease. Enucleation free survival was defined as the time to enucleation from initial eye plaque placement. Time to death and time to recurrence were calculated using the product limit method by Kaplan–Meier, with patients censored at time of last follow-up visit or death.

All 25 custom loaded plaque patients and 20 fully loaded plaque patients had information available regarding tumor characteristics such as maximal tumor apical height (mm), maximal radial diameter (mm), portion of retinal surface (%) and approximate volume of tumor (cc). For all 45 plaques, the total dose (Gy) to tumor apex, lens, sclera, macula and optic disk and dwell time (hr) were recorded. Data was analyzed by treatment group using t-tests with value of *p* <0.05 considered to be significant.

Data involving toxicity outcomes including visual acuity, radiation retinopathy and cataract development were available for all 25 custom plaque patients and 20 fully loaded plaque patients with differing duration of follow-up. To assess for changes in visual acuity, treated patients were subdivided into predefined groups (20/20 up to 20/40, 20/40 up to 20/200 and 20/200 or worse) based on pre-implant and post-implant visual acuity in the affected eye. Movement between these visual acuity groups was characterized as clinically significant visual loss. Additionally, patients were analyzed for visual acuity better than 20/200 whose visual acuity decreased to 20/200 or worse following treatment. 20/200 was chosen due to its definition of legal blindness within the United States per the Social Security Administration. Radiation retinopathy was analyzed as a dichotomous variable based on evidence of retinopathy on ophthalmoscopic fundus examination for all patients and angiography in some cases as well per physician’s clinical judgment. Three patients in the custom plaque cohort and four patients in the fully loaded plaque cohort had a prior history of cataract removal and therefore were excluded from analysis. Therefore 23 of 25 and 16 of 20 patients in the custom and fully loaded plaque cohorts, respectively, were assessed for post-operative development of clinically significant cataracts in the treated eye. Comparison of toxicities was performed using a chi-square test with value of *p <* 0.05 deemed to be significant. All statistics were calculated using SAS software (version 9.4, SAS, Inc., Cary NC).

## 3. Results

### 3.1. Patient characteristics

A total of 20 patients with uveal melanoma who underwent brachytherapy with placement of a fully loaded I-125 eye plaque met our selection criteria and were compared to 25 patients who underwent brachytherapy with a custom loaded I-125 eye plaque (Table 1). 14 patients with custom loaded eye plaques and nine patients with fully loaded eye plaques were male, while 11 patients in each treatment group were female (*χ*^2^ = 0.463). Average age did not differ between groups with a median of 64.0 years and 66.4 years respectively, in the custom loaded and fully loaded cohorts (*p* = 0.529). Each patient had one affected eye and received one implant. 16 patients in the custom loaded cohort and 11 patients in the fully loaded cohort presented with disease in the right eye, while 9 patients in each respective group presented with disease in the left eye (*χ*^2^ = 0.540).

**Table 1:** Tumor characteristics and plaque dosimetric parameters.

Parameter	Median value (range)		Significance
	Custom loaded eye plaques (*n* = 25)	Fully loaded eye plaques (*n* = 20)	
Age (yrs)	64.0 (32–99)	66.4 (53–86)	*p* = 0.529
Sex			
Male	14	9	*χ*^2^ = 0.463
Female	11	11	
Laterality			
Left	9	9	*χ*^2^ = 0.540
Right	16	11	
Tumor thickness (mm)	2.59 (1.75–4.60)	2.78 (2.00–4.00)	*p* = 0.400
Longest dimension (mm)	6.38 (3.50–9.00)	7.12 (4.50–8.50)	*p* = 0.079
Surface area (mm^2^)	28.37 (8.20–62.04)	35.12 (15.00–51.51)	*p* = 0.080
Portion of retinal surface (%)	1.94 (0.55–4.20)	2.37 (1.01–3.48)	*p* = 0.103
Approximate volume of tumor (cc)	0.025 (0.01–0.07)	0.034 (0.01–0.05)	*p* = 0.061
Plaque size			
COMS 10	0	1	*χ*^2^ < 0.0001
COMS 12	0	12	
COMS 12 N	0	4	
COMS 14	10	1	
COMS 14 N	6	2	
COMS 15	1	0	
COMS 16	5	0	
COMS 16 N	3	0	
Muscle disinserted during procedure	15	12	*χ*^2^ = 1.000
Total dose tumor apex (Gy)	79.86 (77.51–80.06)	80.01 (79.94–80.09)	*p* = 0.205
Total dose opposite retina (Gy)	3.43 (2.33–6.10)	3.66 (2.70–4.67)	*p* = 0.328
Total dose sclera (Gy)	187.5 (113.0–356.9)	193.3 (151.1–272.2)	*p* = 0.696
Total dose lens (Gy)	5.53 (3.57–8.82)	5.98 (4.52–8.73)	*p* = 0.251
Total dose macula (Gy)	72.91 (20.30–252.7)	72.32 (12.09–193.2)	*p* = 0.972
Dwell time	96.41 (93.90–100.0)	98.79 (92.50–101.0)	*p* = 0.001
Duration of follow-up (months)	24.00 (12.00–51.00)	60.70 (12.00–97.00)	*p* < 0.0001

### 3.2. Tumor characteristics

Maximum tumor height averaged 2.59 mm in patients receiving custom loaded eye plaques and 2.78 mm in patients undergoing fully loaded eye plaque (*p* = 0.400). Longest dimension average was 6.38 mm in the custom plaque group and 7.12 mm in the fully loaded plaque group (*p* = 0.079). Average surface area of the custom plaque group was 28.37 mm2 and was 35.12 mm2 in the fully loaded group (*p* = 0.080). The portion of the involved retinal surface averaged 1.94% in the custom loaded eye plaques and 2.37% in the fully loaded eye plaque (*p* = 0.103). The approximate tumor volume was on average 0.025 cc in the custom loaded plaques and 0.034 cc in the fully loaded plaques (*p* = 0.061).

### 3.3. Treatment

**Figure 1:**
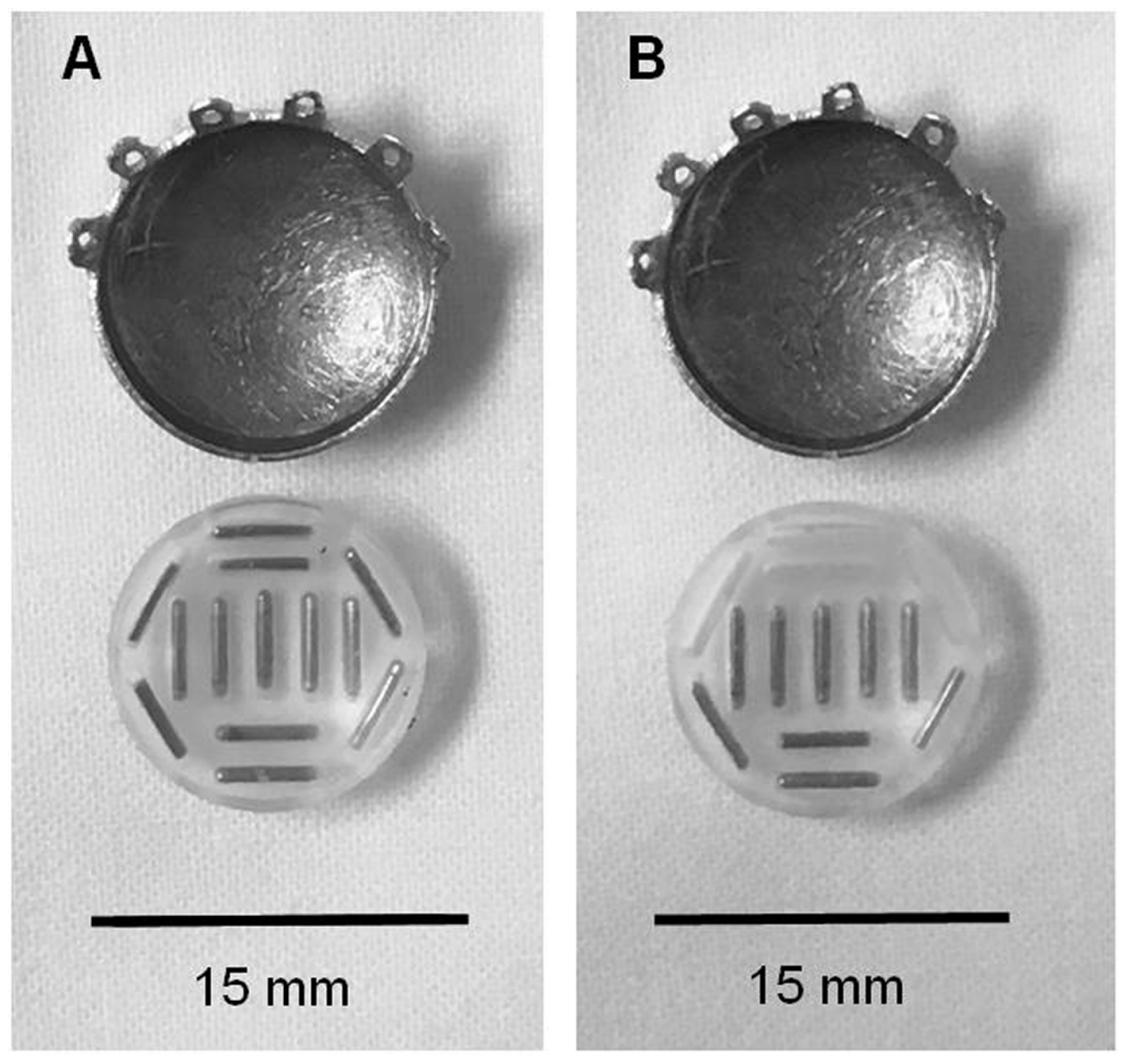
Comparison of fully loaded and partially loaded COMS plaques. **(A)** COMS 14 eye plaque with fully loaded silastic carrier. **(B)** COMS 14 eye plaque with partially loaded silastic carrier.

Commercially available standardized COMS plaques were used in both cohorts. Each plaque had a soft silastic seed carrier cemented to the gold alloy with evenly placed slots for I-125 seed placement allowing 1 mm of carrier between source and sclera. For the fully loaded plaques, each slot was filled with a radioactive Iodine-125 seed. Custom loaded plaques were preferentially loaded in a predetermined area of the plaque allowing for a 2 mm margin around the tumor (see Figure 1). Dose of 80 Gy was planned to the prescription point.

Plaque sizes of 10, 12, 14, and 16 mm as well as notched plaques of size 12, 14, and 16 were used in planning. The distribution of plaques sizes chosen for use in the custom loaded plaque cohort differed significantly from the fully loaded eye plaques (*χ*^2^ < 0.0001). This is an expected outcome as cohorts were matched based on tumor size and larger plaque size is necessary for partial loaded plaques to cover a comparable area. Sizes of fully loaded plaques included 10 mm (1 patient), 12 mm (12 patients), 12 mm notched (4 patients), 14 mm (1 patient) and 14 mm notched (2 patients). Sizes used for custom plaques included 14 mm (10 patients), 14 mm notched (6 patients), 15 mm (1 patient), 16 mm (5 patients) and 16 mm notched (3 patients).

Plaques were placed under monitored anesthesia care in an operating room by one of two staff ophthalmologists (ZC, JA). During the procedure, 15 of 25 (60%) patients receiving treatment with a custom loaded plaque and 12 of 20 (60%) patients receiving treatment with a fully loaded plaque required disinsertion of one of the rectus muscles to assist in plaque placement (*χ*^2^ = 1.000). Plaques were removed after a median dwell time of 96.41 h in custom plaques and 98.79 h in fully loaded plaques (*p* = 0.001). This difference is attributable to a departmental change from planned 100 h dwell times to 96 h dwell times due to operating room availability.

### 3.4. Disease control

Median ophthalmologic follow-up for patients treated with custom loaded plaques was 24.0 months and 60.7 months for those treated with fully loaded plaques. There were no cancer related deaths or deaths due to other causes in either group. Additionally, as of most recent follow-up there were no instances of tumor progression or disease recurrence in either the custom plaque group or the fully loaded plaque groups. Finally, no patients in either group required enucleation due to disease or toxicity and therefore enucleation free survival was 100%.

### 3.5. Toxicity related outcomes

13 patients (52%) who underwent treatment with a custom eye plaque and 14 (70%) patients receiving treatment with a fully loaded plaque developed radiation related retinopathy (*χ*^2^ = 0.2207; Table 2). 39 patients without prior history of cataract surgery were evaluated for the development of a cataract post-operatively. Of those 39 patients, 14 treated with a custom plaque and 10 treated with a fully loaded plaque developed cataracts in the treated eye during the follow-up period (*χ*^2^ = 0.7609). Four patients treated with custom plaques and 11 patients treated with fully loaded plaques were found to have significant visual loss at time of last follow-up (*χ*^2^ = 0.0058). Additionally, four patients (16%) treated with custom plaques and 11 patients (55%) treated with fully loaded plaques were noted to have visual acuity decrease post-operatively to less than or equal to 20/200 (*χ*^2^ = 0.0058).

**Table 2:** Toxicity outcomes.

Parameter	Number of patients (%)		Significance
	Custom loaded eye plaques (*n* = 25)	Fully loaded eye plaques (*n* = 20)	
Toxicities reported at most recent follow-up	Mean follow-up 24.0 months	Mean follow-up 70.2 months	
Clinically significant visual loss	4 (16%)	11 (55%)	*χ*^2^ = 0.0058
Decrease in post operative visual acuity ≤20/200	4 (16%)	11 (55%)	*χ*^2^ = 0.0058
Post-operative cataract development	14 (56%)	10 (50%)	*χ*^2^ = 0.7609
Radiation retinopathy	13 (52%)	14 (70%)	*χ*^2^ = 0.2207
Toxicites reported at 2 Year follow-Up	Mean follow-up 23.2 months	Mean follow-up 24.5 months	
Clinically significant visual loss	4 (16%)	11 (55%)	*χ*^2^ = 0.0058
Decrease in post operative visual Acuity ≤20/200	4 (16%)	9 (45%)	*χ*^2^ = 0.0357
Post-operative cataract development	13 (52%)	5 (25%)	*χ*^2^ = 0.1841
Radiation retinopathy	10 (40%)	9 (45%)	*χ*^2^ = 0.7358

## 4. Discussion

Uveal melanoma is a relatively rare malignancy that can be treated effectively with episcleral brachytherapy (14). Historically all slots of the seed carrier were filled with radioactive I-125 seeds, however a custom loading technique implemented at our institution in June 2013 allows for pre-planned partial seed placement without losing the dosimetric benefits of the silastic carrier. This study directly compares the two techniques in terms of radiation characteristics, treatment outcomes and toxicity related outcomes.

Our first study objective was to compare treatment outcomes of the two groups to assure that treatment with partially loaded plaques did not result in worse outcomes. With a mean follow-up of approximately 2 years for our custom plaque cohort and 5 years for our fully loaded plaque cohort, the two cohorts show no difference in regards to overall survival, progression free survival and enucleation-free survival. In fact, all patients remained without evidence of disease progression both locally and distally and all patients were alive at time of most recent follow-up. This was a higher than expected survival rate for our fully loaded cohort as compared to previously published COMS data which report a 5 year all-cause mortality of 19% in the brachytherapy arm and 10% 5 year mortality rate with histopathologically confirmed metastatic melanoma (14). One explanation for this difference between the COMS study and our fully loaded eye plaques is a difference in tumor size. Our patients had smaller tumor dimensions, on average, when compared to the COMS trial in which the average longest basal dimension was 11.4 mm versus our cohort average of 6.33 mm and 7.12 mm for custom and fully loaded plaques, respectively. As longest tumor basal dimension has independently and statistically significant effects on survival with metastatic melanoma, we expected that both of our treatment groups would have relatively better outcomes in regards to death with metastatic disease (7). In regards to local control, our cohorts once again fared better than historical comparison with a local recurrence rate of 10.3% at 5 years for the COMS study (15). A more recent, larger retrospective multicenter study showed a 4.7% local recurrence at median follow-up of 3.7 years (16). While our outcomes are notably better than these historical controls, taking into consideration the differences in tumor size we feel that our single institutional results are comparable to the historical data.

The second objective of this study was to evaluate rate of treatment related toxicity in our custom eye plaque cohort as compared to our fully loaded plaque cohort; with additional comparison to historical controls. We found no statistical differences in cataract formation between our two cohorts (53.8% for custom plaques at an average of 2 years follow up and 50% for fully loaded plaques at 5 years follow-up). This rate is slightly lower but comparable to the COMS study in which 68% of patients developed cataracts in the first 5 years of follow-up (9). There were also similar rates of development of radiation retinopathy between our two cohorts. This parallels the findings from Gunduz et al. who reported development of non-proliferative radiation retinopathy of 42% at 5 years and proliferative retinopathy rate of 8% at 5 years for posterior tumors (17). Other reports also show similar radiation retinopathy outcomes including 49% at median 74 months (18). Given the discrepancy in follow-up interval we also examined the incidence of treatment related toxicity at 2 years and found similar outcomes (Table 2).

Notably, our study did show a benefit in favor of custom loaded eye plaque in regards to clinically significant visual loss. While the shorter follow-up in the custom plaque group is a possible cause for the difference, it should be noted that our 19.2% instance of clinically significant visual loss is less than historical comparison at a similar time period. Even with use of more severe visual criteria (6 or more lines of visual acuity loss), COMS reported a much a higher incidence of 34% at 2 years (10). As an additional assessment of visual acuity, we computed the percentage of patients with visual acuity of better than 20/200 in whom visual acuity worsened to 20/200 or below post-operatively. Results between our cohorts were significantly in favor of custom eye plaques; at 2 years 15.4% of custom eye-plaque receiving patients had a drop in visual acuity to below 20/200 in comparison to 55% of patients with fully loaded plaques. This is slightly higher than, but comparable to, COMS data of 43% at 3 years and 33% at 2 years follow-up. We suspect that our improved outcomes for visual acuity are attributable to a decreased integral dose to the macula. This is not reflected in our dose to the macula, which is calculated as a point dose and therefore showed no significant difference between the two cohorts (*p* = 0.934).

Due to the retrospective nature of this comparison, there are limitations to our analysis and subsequent conclusions. Chart review was used to obtain outcomes and toxicity data, which has inherent limitations. While these limitations are mitigated by the fact that the majority of patients were diligently followed in house by the same two staff ophthalmologists, a small fraction of evaluations were done at outside facilities due to long travel distance. We also acknowledge that there are many definitions of visual acuity as well as alternative ways to define clinically significant visual loss. Our utilization of a narrow definition is a potential limitation of this analysis. Additionally, we are presenting our early outcomes with an average duration of follow-up of 24 months for our custom plaque cohort. When available, toxicities were compared with historical controls at similar time points to reduce the risk of skewed comparisons, however this was not available in all cases. Finally, due to the rarity of small, posterior uveal melanomas our sample size is relatively small in nature.

In conclusion, our data shows that early outcomes using a novel custom eye plaque were equivalent in terms of survival and recurrence when directly compared to patients treated with fully loaded eye plaques. Additionally, rates of radiation related toxicities including cataracts and radiation retinopathy were found to be similar between patients treated with fully loaded and partially loaded plaques. There also appears to be an early advantage in treatment with partial plaques in regards to clinically significant visual loss and decrease in post-operative visual acuity to worse than 20/200. Finally, the simplicity of this method, in that it does not require a complex I-125 seed inventory or additional technical expertise, makes it applicable to more institutions. Limitations to our study certainly exist and as such we encourage further exploration with prospective direct comparative trials and long-term follow-up. Despite this we remain enthusiastic about the preservation of useful vision demonstrated and support the use and further exploration of partially loaded eye plaques for the treatment of small, posterior uveal melanomas.

## Data availability statement

The raw data supporting the conclusions of this article will be made available by the authors, without undue reservation.

## Ethics statement

The studies involving human participants were reviewed and approved by the University of Cincinnati IRB. Written informed consent for participation was not required for this study in accordance with the national legislation and the institutional requirements.

## Author contributions

SM wrote the IRB and initial draft of manuscript and compiled the data. BH worked with the primary author to come up with the goals and objectives, reviewed the data and results, and edited the manuscript. ZC provided the input on goals and objectives, reviewed the data for inaccuracies, and edited the manuscript. VT edited the IRB and manuscript, reviewed the data and results, and provided the guidance during the entire process and submission. All authors contributed to the article and approved the submitted version.

## Conflict of interest

The authors declare that the research was conducted in the absence of any commercial or financial relationships that could be construed as a potential conflict of interest.

## Publisher’s note

All claims expressed in this article are solely those of the authors and do not necessarily represent those of their affiliated organizations, or those of the publisher, the editors and the reviewers. Any product that may be evaluated in this article, or claim that may be made by its manufacturer, is not guaranteed or endorsed by the publisher.
